# Brain microRNAs differentially expressed in age‐related cerebral pathologies

**DOI:** 10.1002/alz.084740

**Published:** 2025-01-03

**Authors:** Tianze Luo, Selina M. Vattathil, Julie A. Schneider, David A. Bennett, Thomas S. Wingo, Aliza P. Wingo

**Affiliations:** ^1^ Emory University, Atlanta, GA USA; ^2^ Emory University School of Medicine, Atlanta, GA USA; ^3^ Rush Alzheimer’s Disease Center, Rush University Medical Center, Chicago, IL USA; ^4^ Rush Alzheimer's Disease Center, Rush University Medical Center, Chicago, IL USA; ^5^ Emory Goizueta Alzheimer’s Disease Research Center, Atlanta, GA USA; ^6^ Atlanta VA Medical Center, Decatur, GA USA

## Abstract

**Background:**

MicroRNAs (miRNAs) are important post‐transcriptional regulators of gene expression, but we have limited insight into their role in age‐related cerebral pathologies. Here, we investigated the association between miRNAs and nine age‐related cerebral pathologies in participants of the ROS/MAP cohorts.

**Method:**

MiRNA sequencing was performed on samples from the dorsolateral prefrontal cortex of 617 brain donors from participants of the ROS/MAP cohorts. Following quality control, miRNAs were filtered to 528 verified miRNAs, which were included in the global miRNA differential expression (DE) analysis. A genome‐wide miRNA DE analysis was performed for global Alzheimer’s disease (AD) pathology, Lewy body pathology, TDP‐43, hippocampal sclerosis, arteriolosclerosis, cerebral amyloid angiopathy, cerebral atherosclerosis, gross cerebral infarct, and microinfarct. Two models were tested for each outcome: first, a minimally adjusted model adjusting for potential confounding factors, and a fully adjusted model that further adjusted for co‐existing cerebral pathologies to identify independently associated miRNAs. Gene set enrichment analysis (GSEA) was performed on the targets of DE miRNAs to identify the putative biological pathways underlying these pathologies.

**Result:**

With minimally adjusted models, we found DE miRNAs in global AD pathology, Lewy body pathology, arteriolosclerosis, and cerebral amyloid angiopathy. Extending the analysis to adjust for co‐existing cerebral pathologies, we identified 75 independently associated miRNAs for global AD pathology, 45 for Lewy body pathology, 3 for arteriolosclerosis, and 1 for cerebral amyloid angiopathy at FDR < 0.05. Interestingly, we found miRNAs that are shared between AD hallmark pathologies and other age‐related cerebral pathologies. GSEA of target genes of the independently associated DE miRNAs identified biological pathways related to glutathione metabolism, synaptic functions, cellular transport, and innate immune responses altered in arteriolosclerosis at FDR < 0.05.

**Conclusion:**

We identified DE miRNAs in multiple age‐related cerebral pathologies. Our study unveiled shared DE miRNAs between AD hallmark pathologies and other age‐related cerebral pathologies. GSEA results informed putative biological pathways altered in arteriolosclerosis. Collectively, our findings shed light on the role of miRNAs in age‐related cerebral pathologies and establish a basis for future mechanistic studies.

